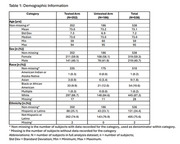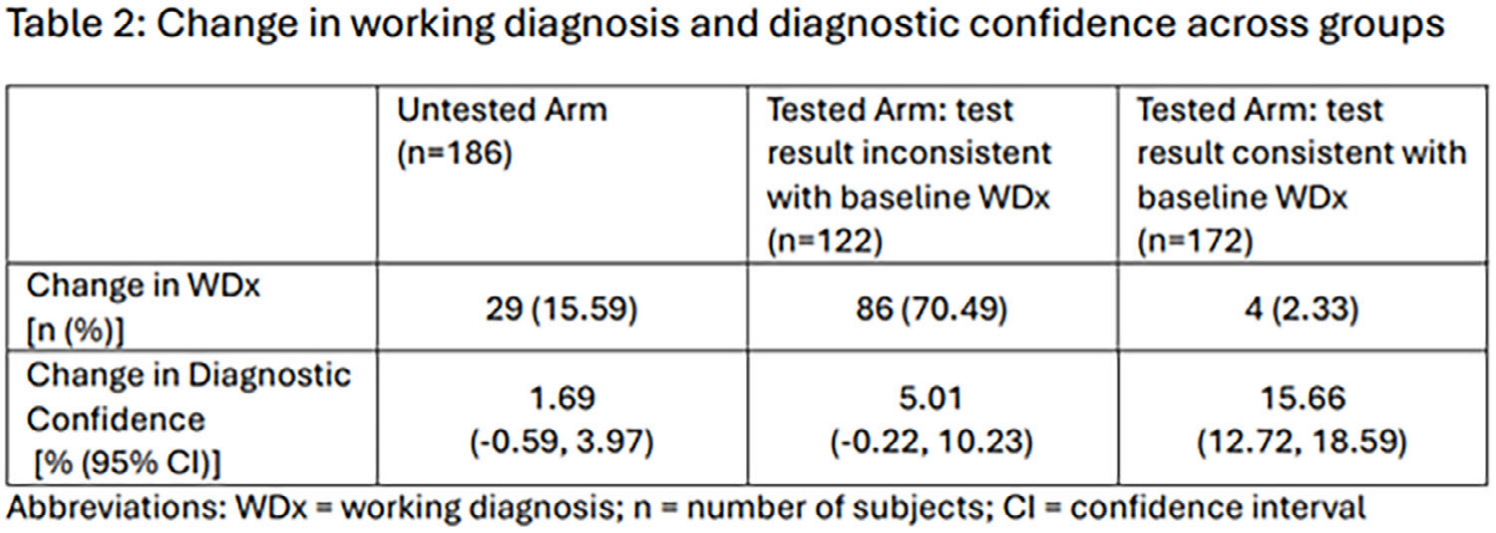# Impact of the Lilly SP‐X *p*‐tau217 plasma assay on management of patients with cognitive impairment: results of a clinical utility study in an ethnically and racially representative cohort, Lilly J4Y‐MC‐B002

**DOI:** 10.1002/alz70856_104863

**Published:** 2026-01-07

**Authors:** Samantha C. Burnham, Haoyan B Hu, Yifeng Tang, Anthony Sireci, Michael Pontecorvo, Rose C. Beck

**Affiliations:** ^1^ Eli Lilly and Company, Indianapolis, IN, USA

## Abstract

**Background:**

Diagnostic criteria for Alzheimer's disease (AD) include confirmation of amyloid pathology. The Lilly SP‐X *p*‐tau217 plasma assay presents a minimally invasive biomarker to identify amyloid pathology; however, clinical utility of plasma biomarkers for patients presenting with cognitive impairment (CI) is not fully understood. This study examined the impact of *p*‐tau217 results on a physician's intended management and confidence in working diagnosis (WDx) for patients evaluated for CI.

**Method:**

A 6‐month, open‐label, randomized, 2‐arm, multicenter, US, prospective observational study of 538 patients presenting for initial evaluation of CI was conducted at 28 sites. Patients were randomized to *p*‐tau217 tested (352) and untested (186) arms. Using questionnaires, WDx (likely AD vs not likely AD), confidence in WDx and intended management (diagnostic, treatment and counselling plans) were obtained at baseline (after initial patient evaluation and prior to testing) and again 4 weeks later. Within the 4‐week period the tested arm received *p*‐tau217 testing; no other new diagnostic results were reviewed. Difference between pre‐ and post‐P‐tau217 results as well as between tested and untested arms for WDx, diagnostic confidence and management plans were evaluated. Data collection for 6‐month actual patient management is ongoing.

**Result:**

Demographically (Table 1), 10.6% identified as Black/African American, 1.8% as Asian and 24.4% as Hispanic/Latino, representative of US population. 85.8% of tested patients had a change in planned management. Where the *p*‐tau217 result (positive or negative) was inconsistent with baseline WDx, a change in WDx was observed in 70.5% of patients, compared to 15.6% in untested patients and 2.3% in those with test results consistent with baseline WDx (Table 2). Where the result was consistent with baseline WDx, a significant (*p* <0.0001) 15.7% increase in diagnostic confidence was observed, compared to 1.7% in untested patients and 5.0% when the result was inconsistent with baseline WDx.

**Conclusion:**

These findings demonstrate clinical utility of *p*‐tau217 testing in a representative sample of the US population being evaluated for CI, specifically in increasing confidence in AD diagnosis and changing a physician's intended management. They also provide evidence for routine use of plasma *p*‐tau217 to support simple, accessible and timely diagnosis, leading to more appropriate patient management.